# Supplementary material for: Human Muse cells reduce myocardial infarct size and improve cardiac function without causing arrythmias in a swine model of acute myocardial infarction
Source: PLoS One. 2022 Mar 24;17(3):e0265347. doi: 10.1371/journal.pone.0265347 (PMC8947423; doi:10.1371/journal.pone.0265347)
Supplement: S1 File — (DOCX) [file pone.0265347.s001.docx]

**Supplementary Tables**

**Data availability (minimal data set)**
